# Dental Ethics

**Published:** 1869-10

**Authors:** H. L. Sage

**Affiliations:** Bridgeport, Conn.


THE
DENTAL REGISTER.

Vol. XXIII.] OCTOBER, 1869. [No. 10.

### Original Communications.

### DENTAL ETHICS.

BY H. L. SAGE, BRIDGEPORT, CONN.

As the code of ethics adopted by the American Dental Association at its sixth annual meeting is the one by which all societies entitled to representation in that body are governed, it follows, that there ought to be a clear apprehension of the ideas embodied in the several articles therein set forth; especially so, from the fact that divers members of the profession guilty of the breach thereof, have plead, in extenuation, an unintentional ignorance or misconception of what constituted a violation.

Whatever may be the opinion of individuals, as to the wisdom or propriety of the Association in adopting such a code, or of its expediency or general effect, it is a fact which most will admit, that until repealed by that body, it should be rigidly enforced by the Association, and practically acquiesced in, and kept inviolate in spirit and in letter by every individual member thereof. Though it may be true, as stated by a member of the committee authorized to draft it, that such a code is " unnecessary for gentlemen, and its enforcement impracticable upon those who are not," it still has this advantage, that it may, as a means of *discipline* for the latter class, prove quite beneficial.

None but gentlemen would add dignity or worth to the American Dental Association, or to any state or local society, by their presence or influence, either as delegates or members; and what would be more effectual in preventing the advent of exceptionable characters into our various Dental societies, or *if* admitted in acting as a restraint upon the natural tendencies of such to the commission of overt acts, or as a check upon *unprofessional* conduct, than a well enforced code of ethics?

With it as a controlling influence, no society need be disgraced by the presence of such. And if, as has sometimes been the case, Dental practitioners of no mean pretensions, of whom we had expected better things, have so far forgotten their obligations, and the relations they have sustained to the profession at large, as to violate its plainest and most reasonable provisions, can it be expected that others of less experience and fewer advantages, and in whom less confidence has been reposed, should not need its restraining influences? Guilt is imputed relatively, in proportion to the intelligence of the guilty party, or the light and knowledge sinned against, and should be so regarded.

Would it have been amiss had an exposition been given by the committee appointed to prepare the code of ethics, of the true intent and meaning of the various points therein embodied, so that none could violate its restrictions under the plea of ignorance of its meaning? As no such explanation has, that I am aware of, appeared in any of the Dental journals, would it indicate a want of modesty should the writer present a few views in relation thereto? Good may result by the comparison, and if unwarrantable inferences are drawn, or wrong conclusions arrived at, light from other quarters may be elicited.

Concerning the first article and its three sections, which treats of the "duties of the profession to their patients," it is, doubtless, clear to the minds of most, what its scope and intent is. Therefore, a few observations will suffice.

Nothing is, unfortunately for the growth of our profession in true worth, more apparent, than the fact that our patients "are, in most cases, unable to correctly estimate the character of our operations." It is often the case, that the most pains-taking efforts which combine to produce the most perfect results, are not at all appreciated by the recipient. Hence, when we find one who does value our endeavors to properly perform our professional duties towards him, we feel more than compensated for our trouble, aside from any pecuniary considerations, and take a just pride in our progress toward perfection. If we act conscientiously and impartially, not permitting any operation to be declared complete until our skill has been exhausted, and the patient has received the benefit of our best services, unless prevented by him from observing such a course, we shall carry out the spirit of that portion of Dental ethics, which treats of the duties of the Dentist to his patients so far as Dental practice is concerned.

But while the former has a duty to perform towards the latter, it is clear that the majority of the public do not, and never will recognize their indebtedness to the Dentist, or in what their duties towards him consists, until his status has been made apparent by the protection of state laws, which shall discriminate for them, who is justly entitled to their confidence and support; and until it is, he must remain content upon an equality, real in some respects and but seemingly so in others, with the unprincipled, incompetent and self-conceited quack.

The second article is composed of requirements, relative to the maintenance of professional character, and is, in reality, but a continuation of the first, one being essentially and practically dependent upon the other in the result to be attained. Many of the points in this article are so plain that comment would be superfluous. Others may appear somewhat ambiguous as to their full meaning, that is, cases may arise in which he may honestly entertain doubts as to how far propriety will allow us to go in certain directions and not conflict with our duty; and as the violation of the third section of article second has proved a more positive base of action against individuals than that of the others, probably because of the greater publicity of some of the acts therein specified, I propose to pay it some attention, but not until first glancing at the preceding sections.

And first, how may a member of the Dental profession maintain its honor? Answer, by endeavoring to maintain his own honor and self-respect. He who respects himself will be honored and respected by others, for self-respect is begotten of those thoughts, desires and affections, which eventuate in good deeds; and he can not respect himself without his motives are so evenly balanced by corresponding acts, as to elevate him from day to day to higher planes of duty and nobler experiences, so that his progress shall be continually apparent to himself, to others and to God. It is only by his own enlargement and growth in moral and intellectual qualities and attainments that he can hope to "extend the sphere of usefulness" of his profession. To be able to impart, he must first receive, and then he must have the disposition to labor for others. "Freely ye have received, freely give."

With such an individual basis of action, the profession at large can not fail to receive benefit therefrom, and ways and means of carrying out liberal designs, and maintaining the honor and efficacy of the profession will naturally become manifest; and it will result in purity of language and conduct, and a respect for the wise and good of the Dental and other professions and avocations. The aged and the young must alike commend themselves by their deportment if they would entitle themselves to respect; and the encouragement of the former will be extended to the latter in proportion to the success of efforts put forth in the face of contending obstacles.

Let me next draw a few references from section 2, article second:

Show me one who is striving with all his power for the mastery of evil, and I will show you a gentleman. Purity within, begets purity without, and cleanliness is akin to Godliness. The latter suggests the former. Not that it is not possible to "make clean the outside of the cap and platter," while neglecting matters equally as weighty, but the weightiest matters must not be passed by, although personal and office arrangements indicative of purity of taste, and a desire for the comfort of his patients, should be sought for by the Dentist, and will be appreciated by the refined and high-toned public; while those of opposite and groveling tastes can not but be favorably impressed, and may be led to seek for higher and better things.

The third section holds up to view, and specifies *unprofessional* acts, and advertising receives a due share of attention, and under this head makes plain what, in this respect, would constitute a breach of the observance of the spirit and letter of the code of Dental ethics. "To resort to public advertisements, cards, hand bills, posters, or signs, calling attention to peculiar styles of work, lowness of prices, modes of operating," is unprofessional. This is a plain statement, and seemingly no one, unless fearfully blinded, need mistake its import. A violation of this portion of the code comes to our knowledge more frequently than any of the others, it being of such public note that "he who runs may read." As the proof is as positive as its publicity, it can not be gainsayed, and the accused can only plead guilty to the charge, unintentional, though the commission of the act may have been. If he seeks an excuse, he will perhaps put it on the ground of a misapprehension of what a violation implies, or of his inability to determine where the dividing line is between what would be considered as proper or improper matter for a public notice of his specialty. Hence, if disinclined to abide by the requirements of the code, he may, perhaps, under such pretense, immediately proceed to make known through the columns of the "Bulletin," of his "ability to perform all operations entrusted to his care at prices lower than the lowest, and cheaper than the cheapest," at the same time "sparing no *pains* to give satisfaction." Another has "the cheapest office in the State," has had "20 years experience" and "warrants all work." Another "inserts an upper or a lower set of teeth for $5.00." Ask him about it, and he will inform you "his only object is to allure; (the simple!) for the best he charges as much as any one." What does this holding out of false inducements indicate? A "moral obliquity of vision"—a letting down of professional dignity and honesty of purpose. But the section under consideration does not, as generally understood, prohibit all kinds of advertising. A handbill with the most modest inscription would not be in correct taste as an advertising medium for a member of the Dental profession in good standing, even though resorted to by traveling medical men of a certain class. Handbills and posters amount to "a distinction without a difference," being so nearly synonymous. A local sign, with name and profession inscribed thereon, would be a legitimate way of calling public attention to location.

The more aristocratic door-plate is not at all prohibited, either by the present version of Dental ethics, or the city authorities. But there is only one more legitimate form to be mentioned as implied by the wording of section three, in which we may invite public attention to our calling. It is the simplest imaginable: a mere business card stating the Dentist's name and location, including the number and name of street, and the name of town or city and state. He who goes beyond this limit, practically ignores the truth of a saying usually accepted as fact, (though a certain and numerous class of the community never act upon it) that "true merit will be sought out," and places himself in the list with him whose assumptions have not always been sustained by corresponding acts, (except in the matter of cheapness, which is a talismanic word with many) and whose merit consists more in his ability to puff himself into notice, than in the possession of attainments, which will of themselves prove a source of attraction to the enlightened portion of the public. But, in the present state of society, the opposite class, constitutes by far the major part, especially in relation to everything that pertains to the profession of Dentistry.

I have not noticed every point in section third, as they are sufficiently plain to need no comment. But that it is unprofessional to "call attention to peculiar styles of work," is a statement requiring some qualification. For example, he who has a valuable article as a base for artificial teeth, should not be restricted from giving it publicity by a modest, succinct and truthful statement of its real merits, which does not necessitate the employment of posters or handbills in the street cars, or other public places, or the usual methods adopted by showmen and others, or the "publishing of certificates" and testimonials for public perusal. Yet, it is now interdicted by the 3d section of article second. "Or go from house to house to solicit or perform operations," is also prohibited, and rightly. There are cases, however, in which it would be no breach of Dental ethics to do this. For an invalid, unable to go out, it would be right and praiseworthy to perform any deeds of mercy pertaining to our profession. Even if called to attend him at his residence—of course this would not be considered out of place.

The first clause of section 4, article second, may claim a passing notice. It reads thus: "when consulted by the patient of another practitioner, the Dentist should guard against inquiries or hints, disparaging to the family Dentist, or calculated to weaken the patient's confidence in him."

We are not to infer from this, that when a case is presented for diagnosis and treatment, we are not to state what the indications are, when known, although it may show improper treatment upon the part of the family Dentist. He whose services are sought by a patient to remedy the bad consequences arising from the mal-practice of a neighboring practitioner, would be doing the latter no injustice, to state to the former wherein the improper treatment consisted, if apparent, and what would have been the right course to pursue, though charity might suggest silence. By no means. So that while we are to look with due allowance upon the blunders of a professional brother, knowing that all are liable to mistakes, at the same time we have our own reputation to sustain, and without enquiring the name of the family Dentist, we may give assurance of our ability to produce the desired result, though it may be an improvement on the practice of our predecessor, provided that ability is ours. To resort to any other course would be doing injustice to the patient as well as the Dentist in charge, for if the case is entrusted to his care he is derelect of duty if he does not give the patient the benefit of his best services, or else decline to operate.

Every Dentist in full practice will, occasionally, be consulted by the dissatisfied patients of others, and requested to give his opinion of the merits, or to point out the defects in a piece of Dental mechanism, for no other purpose than to find some ground upon which to base complaints, which may be brought to bear upon the luckless Dentist who was so favored with his or her confidence, as to be entrusted with the manufacture of a set of teeth which, perhaps, he inadvertently (and unprofessionally too) "warranted to set." In such cases it would be well to employ caution, in expressing an opinion, or, better still, withhold it altogether.

Section 5, which relates to fees, I do not propose to offer any suggestions upon, or upon article 3, concerning the relative duties of Dentists and physicians; and as this paper is already sufficiently extended, the consideration of article 4, which has been somewhat anticipated in other portions of it, will, for the present, be also omitted. Should the exigencies of the case render a further consideration of the subject advisable, it might be resumed at a future day.

